# Analyzing the Explanatory Power of Bionic Systems With the Minimal Cognitive Grid

**DOI:** 10.3389/frobt.2022.888199

**Published:** 2022-05-30

**Authors:** Antonio Lieto

**Affiliations:** ^1^ Dipartimento di Informatica, Università di Torino, Torino, Italy; ^2^ Istituto di Calcolo e Reti ad Alte Prestazioni del Consiglio Nazionale delle Ricerche, ICAR-CNR, Palermo, Italy

**Keywords:** minimal cognitive grid, computational models of mind, computational models of cognition, computational explanation, simulative method, synthetic method

## Abstract

In this article, I argue that the artificial components of hybrid bionic systems do not play a direct explanatory role, i.e., in simulative terms, in the overall context of the systems in which they are embedded in. More precisely, I claim that the internal procedures determining the output of such artificial devices, replacing biological tissues and connected to other biological tissues, cannot be used to directly explain the corresponding mechanisms of the biological component(s) they substitute (and therefore cannot be used to explain the local mechanisms determining an overall biological or cognitive function replicated by such bionic models). I ground this analysis on the use of the Minimal Cognitive Grid (MCG), a novel framework proposed in Lieto (Cognitive design for artificial minds, 2021) to rank the epistemological and explanatory status of biologically and cognitively inspred artificial systems. Despite the lack of such a direct mechanistic explanation from the artificial component, however, I also argue that the hybrid bionic systems can have an indirect explanatory role similar to the one played by some AI systems built by using an overall structural design approach (but including the partial adoption of functional components). In particular, the artificial replacement of part(s) of a biological system can provide i) a local functional account of that part(s) in the context of the overall functioning of the hybrid biological–artificial system and ii) global insights about the structural mechanisms of the biological elements connected to such artificial devices.

## 1 Introduction

Building artificial systems that can exhibit human-like and human-level behavioral capabilities represents one of the main goals of the *two Sciences of the Artificial*, namely, artificial intelligence (AI) and computational cognitive science (CCS) (see Simon, 1980, 2019). While the first discipline, however, is nowadays (in partial contrast with its early scientific ambitions) mainly concerned with only the functional replication of such behavioral capabilities, computational cognitive science (including, the field of computational neuroscience) additionally aims at using such models for explanatory purposes, i.e., to better understand the unknown biological and/or cognitive mechanisms underneath a certain behavior.

From a historical perspective, the latter approach borrows its original inspiration from the methodological apparatus developed by the scholars in cybernetics (Rosenblueth and Wiener, 1945; Wiener, 1961; Cordeschi, 1991). One of underlying ideas of cybernetics was, indeed, one of building mechanical models to simulate the adaptive behavior of natural systems. As indicated in Cordeschi (2002), “the fundamental insight of cybernetics was in the proposal of a unified study of organisms and machines.” In this perspective, the computational simulation of biological processes was assumed to play a central epistemological role in the development and refinement of theories about the elements characterizing the nature of intelligent behavior in natural and artificial systems. The simulative approach was originally inherited by early AI research that used computer programs to reproduce performances which, if observed in human beings, would have been regarded as “intelligent” (see Newell and Simon, 1972), and is also currently used in contemporary bio-robotics (Webb, 2001;Floreano et al., 2014). In a nutshell, the simulative methodology, also known as the synthetic method Cordeschi (2002), proceeds as follows: given a model M of the (cognitive or neural) mechanism hypothesized to produce a certain behavior B by a natural system S, in particular experimental conditions C, the test of the hypothesis assumed in M is obtained by building an artificial system A implementing M and comparing A and S’s behaviors in conditions C. Behavioral similarities between A and S can eventually induce one to conclude that M can produce B in C and that, as a matter of fact, M has an explanatory role with respect to the hypothesized mechanisms used to design A. Otherwise, one may be induced to reject the conjecture. In this article, I argue that within this simulative framework (a typical one in the context of computational cognitive science), hybrid bionic systems enjoy a special status. In particular, the artificial components of such systems (used to replace biological ones) do not offer a direct explanatory role, in simulative terms, about how their internal mechanisms determine a given biological or cognitive function that is assumed to be replaced. I ground this analysis on the use of the Minimal Cognitive Grid (MCG). Despite the lack of such a direct mechanistic explanation, however, I also argue that such systems can have an indirect explanatory role similar to the one played by some structural AI systems partly composed by functional elements. In particular, the artificial replacement of a part of a biological system can provide i) a local functional account of that part in the context of the overall functioning of the hybrid biological–artificial system and ii) global insights about the structural mechanisms of the biological elements connected to such artificial devices. The rest of the article is structured as follows: Section 2 briefly introduces the main design paradigms in the context of computational cognitive science, i.e., the functional and structural ones. Section 3 introduces the Minimal Cognitive Grid (MCG). Section 4 shows, by using two different types of examples borrowed from the literature in hybrid bionic systems, how the use of the MCG outlines the lack of a direct explanatory role, in simulative terms, of the artificial device used in such systems. In addition, I argue that despite the lack of such a direct mechanistic account, the artificial components of the hybrid bionic systems can still play an indirect explanatory role concerning the overall functional and structural elements characterizing the biological system in which they are used. The Conclusions section ends the article.

## 2 Functional vs. Structural Design of Artificial Systems

To better frame the context of simulative methodology in the debate about the explanatory role played by artificial models (and systems) with respect to their analogous in nature, I briefly recall the distinction between functionalist and structuralist design approaches in the context of artificial systems (a distinction partially influenced and borrowed from some theoretical considerations originally started in the philosophy of mind literature).

Functionalism is a concept originally introduced by Hillary Putnam in his seminal article entitled *Minds and Machines* (Putnam, 1960) as a position on the type identity of “mental states.” In this context, mental states are assumed to be understood and characterized on the basis of their functional role. In particular, two tokens are considered to belong to the same mental state if they are in the same functional relation with the other mental states and with the input/output of the system^1^. This approach had a direct influence in AI since, here, it led to the definition of a design approach based on the notion “functional equivalence” between some cognitive faculties and the corresponding mechanisms implemented in the AI programs. Indeed, in such a context, its more radical formulation postulated the sufficiency, from an epistemological perspective, of a weak equivalence (i.e., the equivalence in terms of a functional organization) between the biological/cognitive components and the processes to be modeled, and the corresponding implemented AI components and procedures. In other words, it posited that an explanatory-wise comparison between the behavior of a natural system and an artificial one could have been based purely on the macroscopic equivalence of the functional organization of the constituent elements of the two systems and on their common input–output specifications. This position—at least in its more radical formulation—has been widely criticized in the literature in the last few decades. In particular, models and systems merely designed according to the “functionalist” perspective have been considered not good candidates for providing advances in the science of cognitive AI and cognitive modeling since the mere “functional resemblance” of the components and the mechanisms assembled in the overall design choices adopted in this perspective prevent them from having any kind of direct mechanistic explanatory role with respect to their analogous models available in nature that they aim to model^2^. This is the case, for example, of recent AI technologies, such as some self-proclaimed “cognitive computing” systems like IBM Watson or Alpha Go that despite being the state-of-art systems in their respective fields (and despite showing superhuman performances in the areas on questioning–answering and in the game of Go), *qua* purely functionally designed artificial systems, do not have any explanatory role with respect to how humans solve the same class of problems.

Differently from functionalism, an alternative design approach for building artificial systems whose output can have an explanatory power with respect to the mechanisms used by a natural system to deal with the same problems is the structural approach. Such an approach claims for the need of a stronger constrained connection between the designed artificial systems (and their internal architecture and implemented procedures) and the corresponding ones available in natural biological systems. According to such an approach, structurally constrained artificial models and systems can be useful both to advance the science of AI in terms of technological achievements (e.g., in tasks that are easily solvable for humans but very hard to solve for machines using non-cognitive inspired approaches) and to play the role of “computational experiments,” which could provide insights and results useful to refining or rethinking theoretical aspects concerning the target natural system used as a source of inspiration (Cordeschi, 2002; Milkowski, 2013). Structural systems (often summarized by the expression “function + constraints”) are exactly the class of systems aiming at playing a direct explanatory role in the context of cognitive modeling and computational neuroscience.

Based on this high-level distinction, in the next sections, I present the framework of the Minimal Cognitive Grid, a pragmatic methodological tool proposed in Lieto (2021) to rank the different degrees of structural accuracy of artificial systems in order to project and predict their explanatory power (along the functional–structural continuum) and show how its applications to two different typologies of bionic systems (borrowed from Datteri, 2017) outlines the absence of a direct mechanistic role of these artificial devices substituting biological components in this class of hybrid systems.

## 3 Structural Accuracy of Artificial Systems: The Minimal Cognitive Grid

The notions of both cognitive and biological plausibility, in the context of computational cognitive science and computational modeling, refer to the level of accuracy obtained by the realization of an artificial system, with respect to the corresponding natural mechanisms (and their interactions) that they are assumed to model. In particular, the cognitive and biological plausibility of an artificial system asks for the development of artificial models that are i) consistent (from a cognitive or biological point of view) with the current state-of-the-art knowledge about the modeled phenomenon, and that ii) adequately represent (at different levels of abstraction) the actual mechanisms operating in the target natural system and determining a certain behavior. Some of the key questions to answer in this regard are which are the elements (e.g., the processes, mechanisms, structures, etc.) in the inspiring natural system those enabling the rise of the desired behavior? To what extent does the obtained behavior depend on such elements? In the context of biologically inspired artificial systems, different general criteria have been proposed to characterize the design of biologically plausible models. In this regard, the roboticist Webb (2001) identified the following list of seven dimensions for the characterization of different design aspects of bio-inspired models.

1. “Biological relevance”: this dimension shows if and, eventually to what extent, a computational model can be used to generate and test hypotheses about a given biological system taken as a source of inspiration.
2. “Level”: this dimension aims at individuating “what are the basic elements of the model that have no internal structure or their internal structures are ignored.” In other words, it identifies the modeling focus. For example, an atomic model could be focused on the internal structures of the atoms or could ignore such an issue by focusing on the interactions between atoms (of course, usually the choice of the “level” also determines what class of formalisms can be adopted).
3. “Generality”: this feature aims at individuating how many different biological systems can be represented by a model.
4. “Abstraction”: this dimension considers the amount of details included in the artificial model with respect to the natural system taken as the source of inspiration. According to Webb’s terminology, “abstraction” should not be confused with the “level” dimension. A more abstract model of a cognitive process could indeed contain more details and be more complex than the corresponding lower level brain model of the same mechanism.
5. “Structural accuracy”: this dimension intends to measure the similarity of the mechanisms behind the behavior of an artificial model with respect to those of the target biological system. This aspect is directly affected by the state-of-the-art knowledge of the actual mechanisms in biological systems and is not necessarily proportional to the amount of details included in the model.
6. “Performance match”: this dimension is intended to account for the similarity of the performances of the model with respect to the performances obtained by the target biological system.
7. “Medium”: this dimension refers to the physical medium that has been used to implement the model.

Despite the huge influence of Webb’s characterization for what concerns the dimensions to take into account when designing and evaluating bio-inspired systems, this proposal is, however, limited in a number of ways. First, it explicitly targets only biologically plausible constraints (since, as mentioned, Webb is a roboticist and her interests explicitly target issues dealing with control and physical constraints). It does not consider, for example, different types of higher level cognitive constraints that one could indeed consider in a “plausible” model of human (or animal) cognition^3^. For the same reason, it does not consider non-embodied agents/simulations, thus leaving aside a huge class of models developed within the cognitive modeling and AI communities^4^. Furthermore, some of the dimensions appear to be not self-explanatory. For example, the concept of “biological relevance” or “structural accuracy” are highly overlapping, and there is not a clearly defined method that one could use in order to determine how such elements are/can be operationally characterized. Similarly, the concept of “medium” is assumed to consider the physical instantiation carrying out the computations of the computational model and is evidently related to the physical level in the Marr’s hierarchy (Marr, 1982). However, Webb’s proposal explicitly limits the considerations on this aspect to the presence (or not) of an embodied agent. The “medium,” in her view, is the physical body of the agent (a robot). This view is, however, quite restrictive since it does not consider, for example, alternative physical models of computations based on, for example, hybrid biological/artificial systems realized in the field of bionics and neuromorphic computing (and these are the focus of the current work). Finally, Webb’s proposal provides a powerful, but only intuitive account of the dimensions proposed in order to design and classify different bioinspired models. While this effort is very useful for outlining a sort of *Design Atlas*, it does not fully serve the engineering purpose of providing an operational characterization that can outline and classify, in more precise terms, the differences and similarities of the diverse possible instantiations of biologically or cognitively inspired artificial systems.

In Lieto (2021), by building on Webb’s insights, I proposed a much more synthetic list of elements that subsumes and abstracts some of Webb's dimensions and that, additionally, can not only be applied to hybrid bionic systems but also to classical biologically inspired and cognitively inspired ones. This latter aspect is important since “artificial plausibility” can be obtained at different levels of abstractions (not only at the neurophysiological or biological level) and using different formalisms and modeling approaches. In addition, the proposed characterization has the merit of providing a set of characteristics that can be directly used to compare different biologically or cognitively inspired systems and can be used as a tool to project their explanatory power. The proposed minimal set of analytic dimensions to consider, that I called the “Minimal Cognitive Grid” (MCG), is composed by the following aspects:

“Functional/Structural Ratio”: this dimension concerns the individuation of the elements upon which the artificial model/system is built. For example, in a complex artificial system (embodied or not), it is possible to model in a “functional” way some elements of the system whose internal structures and mechanisms are not considered important with respect to ones' explanatory goals, and on the other hand, it is possible to build structural models of other components of the same system. In other words, this dimension considers the ratio between functional and structural components (and heuristics) considered in the design and implementation of an artificial system. This ratio depends on the actual focus and goal of the model and can be used for both integrated systems performing different types of tasks and for narrow and task-specific systems. This dimension synthesizes and subsumes the “Biological relevance” and “Structural accuracy” individuated by Webb by enabling, in principle, the possibility of performing both a quantitative and qualitative comparison between different cognitively inspired artificial systems. Of course, in this case, the lower the ratio, the better. The “system dissection” required by this dimension of analysis is also useful to individuate the kind of explanations that can be ascribed to the different components of the systems (e.g., a direct mechanistic explanation would typically make sense only for the “structurally modeled” components).

“Generality”: as for Webb’s proposal, this feature aims at evaluating to what extent a given system/architecture can be used in different tasks, i.e., how general is the model and how much of it can be used to simulate a set of biological or cognitive functions and not just a narrow one. Also, this element can be considered both from a quantitative (e.g., by counting how many cognitive faculties can be modeled within a single model/system) and qualitative point of view.

“Performance Match”: as for Webb’s proposal, this dimension involves a direct comparison between natural and artificial systems in terms of the obtained results for specific or general tasks. With respect to Webb’s account, however, I propose a more precise characterization of this dimension. In particular, I suggest taking into account some of the main hints of the psychometric AI movement (Bringsjord, 2011) asking for the use of a battery of validated tests to assess the effective “match” between artificial and biological systems. In this line, thus, I also propose to consider two additional specific requirements that refers to such an aspect: 1) the analysis of the system errors (that should result to be similar to those committed by the biological system considered as the source of inspiration) and 2) the execution time of the tasks (that again should converge toward the performances of the biological system in focus)^5^. Therefore, in this configuration, the degree of accuracy obtained by artificial systems in modeling certain performances is not sufficient to claim any kind of biological or cognitive plausibility. Of course, the inclusion of the two additional requirements also similarly does not guarantee any plausibility claim (since a system could match these additional psychometric measures without being a “structural model”). However, it is worth noticing that all three dimensions conceived for the MCG, considered together, can provide a nonsubjective evaluation of the structural accuracy of a model. As for the first two dimensions, indeed, the rating assumed on this dimension can also be, in principle, determined by both quantitative (e.g., by considering the differences in terms of the results, errors, and execution times between the natural and artificial systems) and qualitative means. In addition, all the three dimensions allow a graded evaluation (i.e., they allow nonbinary yes/no answers but a graded ranking). Finally, the nature of such dimensions allow for a nonsubjective evaluation [differently from other classical tests proposed in AI and cognitive science such as the Turing Test (Machinery, 1950), and its variations, or the Newell test for cognition (Anderson and Lebiere, 2003)^6^]. Table 1 synthesizes the main features characterizing the Minimal Cognitive Grid.

**TABLE 1:** The three dimensions of the Minimal Cognitive Grid individually analyzed with respect to their epistemic goal and the types of allowed evaluations.

	Epistemic goal	Quantitative evaluation	Qualitative evaluation	Graded evaluation	Subjective evaluation
Functional/structural ratio	Evaluating the biological/cognitive adequacy of the artificial system *via* system dissection of its components/mechanisms	Yes	Yes	Yes	No
Generality	Evaluating the transferability of a given system/model to different tasks and biological/cognitive functions	Yes	Yes	Yes	No
Performance match	Comparing the output of the artificial system with the natural one(s) in terms of i) results, ii) errors, and iii) response times	Yes	Yes	Yes	No

Summing up, by starting from the original proposal by Barbara Webb, I individuated a minimal set of dimensions (the “Minimal Cognitive Grid”) that can be used as an analytical tool to compare different kinds of cognitive artificial systems and their degree of structural accuracy with respect to human performances and abilities. This tool is general enough to include both biologically and cognitively inspired modeling approaches and allows comparing them in terms of their explanatory capacity with respect to a natural system taken as the source of inspiration. In addition, it is applicable also to the class of hybrid bionic systems.

## 4 Two Types of Simulations in Hybrid Bionic Systems and Minimal Cognitive Grid

Bionic systems are a well-known class of hybrid artificial systems connecting biological tissues with computers or robotic devices through brain–machine interfaces. These technologies may enable the restoration of communication, sensory and motor abilities lost due to accidents, stroke, or other causes of severe injury (for example, see Hochberg et al., 2006). In addition, leading researchers have claimed that bionics technologies can provide unique and new experimental tools to discover brain mechanisms (e.g., see Chirimuuta, 2013) and that from an epistemological point of view, such systems are assumed to be useful in various ways to discover hidden or unknown aspects of biological mechanisms (see Datteri, 2009; Datteri and Tamburrini, 2007). Based on the distinction proposed in Datteri (2017) between “stimulation–connection” and “simulation–replacement methodology” for the study of the brain I will show in the following how the use of the MCG supports the argument attributing the lack of a direct explanatory account of the artificial components used in such systems.

### 4.1 Stimulation–Connections vs. Simulation–Replacement Systems

Datteri (2017) analyzes two different case studies to introduce the distinction the simulation–replacement methodology (also called ArB “Artificial replacement of Biological components”) from the so-called “stimulation–connection” methodology. The main difference is that the stimulation–connection methodology may assist in the theorization over the biological components connected to the prosthesis (hence the “connection” label), while the simulation–replacement methodology may enable one to model the behavior of the biological component replaced by the prosthesis (hence the “replacement” label). In addition, the simulation–replacement methodology is akin to the “synthetic method” since theoretical results can be drawn from comparisons between the behavior of the target biological system and the behavior of the hybrid system, which can be regarded, in its entirely, as a hybrid simulation of the target hypothesis. Stimulation–connection studies, on the other hand, make a non-simulative use of machine models of biological systems since they apply relatively traditional electrophysiological analysis techniques to neural tissues which are peculiarly stimulated by a connection with an artificial device.

These different classes of bionic models are instantiated in two types of bionic systems used in diverse experiments (borrowed from Datteri, 2017) that we will call the “lamprey experiment” and the “monkey experiment.” The lamprey experiment is an example of the simulation–replacement methodology and consists of the development of a mechanistic model of the lamprey sensory–motor system, obtained by Zelenin et al. (2000) *via* the replacement of the reticulospinal pathway of the lampreys (a portion of the lamprey nervous systems thought to play a crucial role in the lamprey capability of maintaining a stable roll position by moving its tail and other body parts in response to external disturbances) with an electromechanical device. The input–output behavior of such a device corresponds to the hypothesis assumed by all the research performed on the relationship between the “input” neurons of the reticular neurons (RS) and the roll angles of the animal, which vary as a function of the activity of the “output” spinal neurons. The artificial component picked up the activity of the reticular neurons and produced stabilization movements in line with the hypothesized regularity. Zelenin et al., in this way, have experimentally tested whether the hybrid system exhibited stabilization abilities comparable to those of the intact system. As this happens to be the case, the authors have therefore concluded that the electromechanical device was a good substitute for the RS component, and as a consequence, the RS component actually exhibited the hypothesized input–output regularity r (rs). In other words, the comparison in this case has been made purely at the functional level of the input–output behavior of the replaced component with respect to the original replaced biological component.

On the contrary, the “monkey experiment” (and in particular, one of the trials of this study concerning a brain control study done with monkeys) has a different status. I will briefly recall here the whole experiment and then the “brain control” portion that is of interest in this article. Carmena et al. (2003) describe a study where two monkeys chronically implanted with microelectrode arrays in various frontal and parietal brain areas had been trained to perform three kinds of tasks. In the first one, they had to move a cursor displayed on a screen and reach a target by using a handheld pole. In the second one, they had to change the size of the cursor by applying a gripping force to the pole. The third task was a combination of the first two. Neural activity was acquired, filtered, and recorded during the execution of these tasks. Two different uses have been made of these neural recordings in two distinct phases of the study. In the first phase, the monkeys had to move and control the cursor by using a pole. In the second one, the control was demanded to an external device decoding the signals of the implanted brain machine interface. During the first “pole control” phase, a reliable correlation was identified between the neural activity and motor behavior of the monkeys. More precisely, a linear model had been trained to predict various motor parameters—hand position, hand velocity, and gripping force—from brain activity.

**FIGURE 1:**
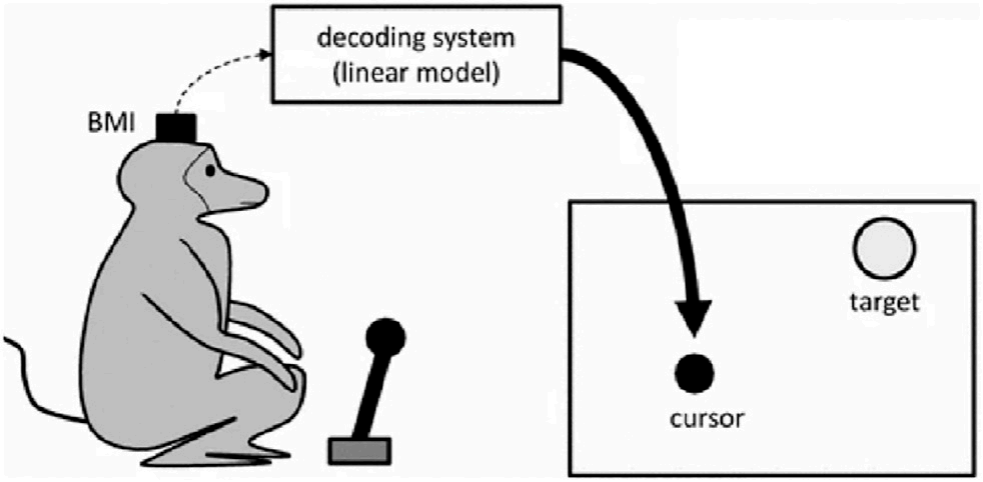
Pictorial representation of one of the experiments described in Carmena et al. (2003), where the cursor is “brain controlled” *via* the decoder.

After obtaining a predictively adequate model, the authors proceeded with a so-called “brain control” phase. During this phase, the cursor positions were controlled not by the pole but by the output of the linear model receiving brain activity as the input (see Figure 1). The monkeys had to carry out the same three tasks, obtaining rewards on successful trials. I will not dwell here on the “pole control” part of the experiment since it represents a classical example of the application of a functional resemblance similar to the one on the lamprey. The brain control experiment, on the other hand, belongs to the class of a “stimulation, non-simulative bionics-supported methodology for the discovery of brain mechanisms” (Datteri, 2017). In this case, in fact, even though an artificial model of the replaced biological component is used, these results are obtained by applying relatively traditional electrophysiological analysis techniques aimed at analyzing the responses of the biological tissues connected to the artificial device and, as a consequence, at understanding or hypothesizing some of their unknown mechanisms.

The main outcome of this experiment is that brain control of the cursor (and, in another trial of the study, of a robotic prosthesis controlling the cursor) is possible. In addition, the authors describe the following outcomes: i) since control performance suddenly declined in the switch between pole and brain control modes, and then progressively improved^7^ even if not reaching the original outcome, they assumed that this result could be explained by assuming that an efficient motor control requires a neural representation of the dynamics of the controlled object (a representation that is absent in the first trial). This hypothesis is supported by other results coming from the analysis of directional tuning (DT) profiles of individual neurons and ensembles in the “brain control” phase. DT profiles model the relationship between neural activity and direction of movement (e.g., by outlining that a particular neuron fires maximally whenever the monkey moves its arm leftward). Such profiles have been recorded during the “pole control” and the “brain control” phases, by modeling the relationship between neural firing and cursor movements and gradual changes in DT profiles have been found after switching from pole to brain control. In particular, at the very beginning of the “brain control” phase, a general decline of DT strength (i.e., of the strength of the correlation between firing activity and movement direction) had been detected. A further decline had been observed when the monkeys ceased to move their limbs. Later on, gradual increases in DT strength have been detected while the monkeys progressively improved their brain-control proficiency, but the levels measured during pole control have never been reached again. According to the authors, these results shed some light on the mechanisms of sensory–motor control in the intact biological system. In particular, they claim that the sudden decrease in DT strength after switching from pole to brain control, and especially the fact that DT strength was low even at the very beginning of the second phase when the monkeys were still moving the pole, suggests that DT profiles do not reflect only movement direction as signaled by proprioception (this kind of feedback was available at the beginning of the “brain control” phase). The successive increases in DT strength, when proprioceptive feedback was totally uninformative of cursor direction, further support the thesis that monkeys’ brains can progressively acquire a neural representation of the movements of the new actuator based on visual feedback only. Thus, they hypothesized that as monkeys learn to formulate a much more abstract strategy to achieve the goal of moving the cursor to a target, without moving their own arms, the dynamics of the robot arm (reflected by the cursor movements) become incorporated into multiple cortical representations. In other words, the authors propose that “the gradual increase in the behavioral performance during brain control of the BMI emerged as a consequence of a plastic reorganization whose main outcome was the assimilation of the dynamics of an artificial actuator into the physiological properties of frontoparietal neurons” (Carmena et al. (2003); p. 205).

### 4.2 Minimal Cognitive Grid Analysis and the Indirect Explanatory Role of Bionic Systems

In the following, I analyze the two abovementioned case studies concerning different types of bionic systems through the lenses of the Minimal Cognitive Grid in order to assess the direct explanatory power of the artificial components used in such systems.

Let us consider the lamprey case study first. In this case, the functional/structural dimension of the MCG cannot be used since the substituted controller is entirely functional in that it simply processes the same input–output specifications of the biological tissue. For what concerns the “generality” criterion, the kind of extracted motor configuration parameters are limited to the substituted part and, as a consequence, the obtained results are not directly generalizable to other components of the biological system. Finally, for what concerns the performance-match, the authors have seen that there is a good correspondence with the functional performances of the biological system (i.e., the behavior is functionally comparable). However, no results are reported concerning the cases of failure. Overall, the MCG shows how this artificial device does not have *per se* a direct mechanistic explanatory power to play (and this, despite the overall methodology used in this experiment still belongs to the class of simulative method since it allows, as we have seen, other kinds of explanatory practices).

Also, for what concerns the brain control experiment, the MCG shows a lack of any direct explanatory power of the artificial component implanted in the monkey’s brain. In particular, there is no structural element used to build such a specific device (that is only aimed at replicating the same behavior of the replaced biological part), and therefore, no functional/structural ratio is applicable. In addition, as in the previous case, it is not possible to assume any level of generality in the control model learnt and used by the artificial device with respect to other biological parts and, finally, the performance-match of the hybrid system with respect to a non-implanted monkey is not carried out since the experiment pursues a different goal other than the performance comparison with a corresponding intact natural system.

Despite the lack of such a direct mechanistic explanation of the artificial component, however, these kinds of hybrid bionic systems can have an indirect explanatory role similar to the one played by some AI systems built by using a structural design approach obtained with the help of some functional components. In particular, the artificial replacement of a part of a biological system can provide i) a local functional account of that part in the context of the overall functioning of the hybrid biological–artificial system, and ii) global insights about the structural mechanisms of the biological elements connected to such artificial devices.

For what concerns the first point, on one hand, the lamprey experiment is paradigmatic for the class of hybrid bionic systems using a functional approach: the functional comparison of the input–output behavior of the replaced component with respect to the original biological one does not suggest any particular insights on the mechanisms (and their eventual biological plausibility) regulating the behavior of the artificial component. It is possible, however, to have a functional account of the substituted part. For what concerns the second point, on the other hand, the brain control experiment on the monkeys is also enlightening. As indicated above, indeed, even if no direct comparison is done with the corresponding biological/intact system, there is a sort of performance comparison done between the brain control and the pole control conditions about the analysis of directional tuning (DT) profiles. In particular, the difference of the two profiles (that can be seen as a sort of error analysis) has led to the development of some explanatory hypothesis about the overall general mechanisms characterizing the organization of the biological tissues connected to the artificial device. This element represents one of the possible indirect explanatory uses that such systems can perform. Despite, as reported in Datteri (2017), the reported conjectures consist in a very large-grained, tentative, and incomplete sketch of the mechanism implemented in the biological system to which the prosthesis is connected, the proposal of such mechanistic hypotheses would have not be possible without the analyzed behavior of the bionic system. From a historical perspective, the emergence of mechanistic hypotheses coming from structural models incorporating some relevant functional components is partly related to some classical examples coming from the simulative methodology in cognitive science. For example, the classical context of well-known cryptarithmetic problems having the form: DONALD + GERALD = ROBERT (Newell and Simon, 1972) and modeled by using symbolic approaches show how such a class of models, which are even structurally inadequate from a neuroscientific point of view, can be, nonethenless, a useful mean to model a computational enquiry to discover a structural hypothesis of our reasoning mechanisms. In this specific case, ten distinct digits had been substituted for the ten distinct letters in such a way that the resulting expression is a correct arithmetic sum (526,485 + 197,485 = 723,970). As the problem is usually posed, the hint is given that D = 5. The symbolic program solving this problem solves the problem, as the humans, by finding the values for the individual letters in a particular sequence: T = 0, E = 9, R = 7, A = 4, and so on …The reason is that only if this order is followed can each value be found definitely without considering possible combinations with the values of the other letters. With this order, in fact, the solver does not have to remember what alternative values he has assigned to other variables, or to back up if he finds that a combination of assignments leads to a contradiction. In other words, the search behavior of the information-processing system derives directly from the system’s small short-term memory capacity. In addition, the empirical fact that human solvers do make the assignments in roughly the same order has provided an important piece of evidence (others can be obtained by analyzing, thinking-aloud protocols, and eye movements) that the human information-processing system operates as a serial system with limited short-term memory. In this case, in fact, the performance of the information processing system matches the verbal protocol. In other words, the symbolic model usually adopted to model this problem does not consider neurological constraints, and as such cannot be considered a structural model of brain processing. The system, in fact, is compared with human solvers in a functional way. However, it makes explicit heuristic assumptions about the algorithmic level of the problem from an information processing perspective (e.g., the constraints about the space and memory limits, the sequential processing, and so on) and, as shown, can be useful to provide structural information about the processing modes and mechanisms of the overall modelled cognitive phenomenon.

As mentioned above, the case of the brain control experiment is partially similar to the classical Newell and Simon’s case of the cryptharithmetic problem. Also in this case, a functional component of the system can provide insights on the structural mechanistic concerning the global behavior of the hybrid system. Differently from that, however, the indirect explanatory role played by the overall bionic system is in the mechanistic hypotheses that can be drawn over the biological components of the system (and not on the artificial one). In the classical simulative methodology, this case is not contemplated and represents, *de facto*, one of the main distinguishing elements of the field of bionic simulation of biological and cognitive faculties. Overall, this outcome confirms and strengthens by using a novel methodological tool, the main insights in Datteri (2017). As a consequence of this state of affairs, the hybrid bionic models seem to have a full room in both biologically inspired AI and biological and cognitive modeling research agendas. In addition, however, if we move away from the analysis of local explanations and look for global explanatory phenomena in this kind of hybrid systems, the MCG additionally allows to project them (and the potential explanatory role that the different strategies they embed can, in principle, play in bionics) along the functional–structural design continuum.

In particular, if we analyze with the MCG, the global explanatory possibilities offered by the two different methodologies analyzed in the presented case studies, we can observe that the functional/structural dimension is applicable to both types of systems. In particular, the structural components of these bionic systems would be represented by the actual biological parts of the overall systems (that do not represent a model of themselves, but actually their very same replica) and the functional components will be represented by the part(s) of the functional artificial devices connected to such components. The generality dimension still seems potentially valid to analyze the explanatory role of both these diverse classes of systems, but in the analyzed case studies, there is no evidence of having discovered or unveiled biological mechanisms generalizable to other biological or cognitive functions. Finally, the performance-match dimension, even if in principle is analyzable for both the systems, is only available for the system of the lamprey case study, conforming to the classical simulative methodology. Overall, the explanatory accounts that we can obtain from the systems presented in this work indicate, in principle, a higher explanatory potential for the lamprey systems than for the brain-controlled one. Tables 2, 3 summarizes this state of affairs. This difference confirms the diverse epistemological status assigned by Datteri (2017) to the simulation *vs*. stimulation-based methodologies.

**TABLE 2:** Synthetic table concerning the analysis with the MCG of the *local* explanatory power of the different bionic systems adopted in the simulation *vs*. stimulation methodologies.

	Functional/structural ratio	Generality	Performance-match
Lamprey study	Not applicable (functional design)	No	Functional replication
Monkey study (brain control)	Not applicable (functional design)	No	No match

**TABLE 3:** Synthetic table concerning the analysis with the MCG of the two *global* explanatory powers of the different bionic systems adopted in the simulation vs. stimulation methodologies.

	Functional/structural ratio	Generality	Performance-match
Simulative methodology lamprey study	Applicable	No	Accuracy performance
Simulative methodology brain control monkey study	Applicable	No	No match

## 5 Concluding Remarks

Summing up, in this article, I have analyzed two different types of hybrid bionic systems that according to the distinction introduced in Datteri (2017), allow to produce different kinds of explanatory hypotheses about the biological mechanisms they model (in a functional or structural fashion). This analysis has been carried out with the help of a new methodological tool called the Minimal Cognitive Grid (MCG). This analysis suggests that different from the classical account of artificial models of cognition, the artificial component of the hybrid bionic system does not play, alone, any direct mechanistic explanatory role concerning its internal biological adequacy (compared to one of the biological tissue replaced) within the overall functioning of the entire system.

Overall, in the literature on the explanatory account of hybrid systems, this conclusion is aligned with the one provided by Craver (2010). This author distinguishes between affordance and validity of a model (defined as “to the extent that the behavior of the simulation could replace the target in the context of a higher-level mechanism” (p. 842). For example, a robotic arm enabling one to perform all the movements and actions that he/she could perform with a biological arm, like grasping, lifting, or pushing objects, is affordance valid); phenomenal validity (defined as “to the extent the input–output function of a model is relevantly similar to the input–output function of a target (natural) system”) (*ibid.*); and mechanistic validity [defined as “to the extent that parts, activities, and organizational features represented in the model are relevantly similar to the parts, activities, and organizational features in the target” (*ibid.*)]. Based on this distinction, Craver claims that hybrid bionic models (which he calls *prosthetic models*) should be affordance valid models. And this status does not imply any phenomenal or mechanistic validity. From the analysis done *via* the MCG, it similarly emerges that phenomenal validity and mechanistic validity do not follow each other and that the behavioral capability of a prosthesis to replace a given biological component of a system (also called *target* in Craver’s terms) does not imply that neither the fact that they share the same input–output mapping, as assumed in the phenomenal validity account^8^, nor any mechanistic validity (since the simulated higher-level mechanisms could be just functionally replaced by the artificial component). On the other hand, however, different from the analysis by Craver, the MCG suggests that, as outlined in Datteri (2017), there is an epistemic difference between the different types of hybrid bionic systems available in the literature. In particular, being a tool designed to detect the direct and explicit design assumptions used in an artificial system, the MCD assigns a higher explanatory potential to the simulative hybrid systems (paradigmatically represented by the lamprey case study) than to the ones built by using a stimulation-based methodology. However, the direct explanatory coverage of MCG also suggests that there is the possibility of some indirect mechanistic explanatory account that can be addressed with such systems in the context of the stimulation-based systems. And that, therefore, is not possible to completely exclude hybrid bionic systems as tools to be used in discovering brain mechanisms. In particular, the kind of functional replacement adopted in such systems can help, in the different analyzed configurations, in providing a functional explanatory account of the overall system behavior or (in the case of stimulation-based methodology) can indirectly support the processes of hypotheses formation, formulation, and testing concerning the mechanisms of the biological elements connected to the artificial component. Overall this outcome confirms and strengthens, by using a novel methodological tool, the main insights in Datteri (2017). As a consequence of this state of affairs, this work suggests that hybrid bionic models can play a crucial role in both biologically inspired AI (not aiming at playing any explanatory role with respect to the corresponding biological systems taken as a source of inspiration) as well as in explanatory-seeking, biological, and cognitive modeling research agendas. While there is a certain agreement on the role played in the first context (Prinz, 2004; Craver, 2010), the insights coming out form this analysis also support the use of such systems in these latter scientific enterprises.

## Data Availability

The original contributions presented in the study are included in the article. Further inquiries can be directed to the corresponding author.

## Footnotes

1 Within the philosophical tradition, functionalism has been proposed in many different forms (for example, Jerry Fodor posed more strict requirements with respect to the functionalist analysis of the mind proposed by Putnam). We will not dwell here on these different proposals widely discussed in the literature on the philosophy of the mind (a complete overview is provided in https://plato.stanford.edu/entries/functionalism/). The main point here is to recognize the influence that this position has had in the context of AI where it has been somehow translated, with different nuances and implications, in the context of practical design issues for building artificial systems.

2 It is worth noticing, however, that such systems still preserve the possibility of types of explanations other than the mechanistic one. For example, their behavior could be explained with functional or teleological explanations. However, in the first case, as indicated by Piccinini (2007) (p. 125) “Computational models that embody functional explanations explain the capacities of a system in terms of its sub-capacities. But this explanation is given by the assumptions embodied in the model, not by the computations performed by the model on the grounds of the assumption”. On the other hand, teleological explanations, despite being important in the context are not exactly what one would expect from a computational model. See Lieto (2021) (chapter 3) on these issues.

3 Examples of such constraints for human cognition can be borrowed from the literature from cognitive, developmental, and experimental psychology. They can concern, for example, very specific aspects like the resource-bound typicality-based strategies used for categorization (Lieto et al., 2017) or more general aspects like the reasoning heuristics for decision making under uncertainty (Gigerenzer and Todd, 1999; Lebiere and Anderson, 2011).

4 This does not mean that many of the Webb’s criteria cannot be equally applied to non-embodied simulations. It means, however, that non-embodied agents/simulations are not explicitly considered in the Webb’s account since her focus has been on bio-robotic models, and she wants “to rule out purely computer-based models (i.e., where the environment as well as the animal is represented in the computer), and also computer sensing systems that terminate in descriptions rather than actions” (Webb, 2001, p. 1037). As mentioned, however, this restriction leaves out from her analysis many biologically plausible simulative models used, for example, in computational neuroscience and computational cognitive science.

5 In principle, the convergence in execution times toward the performances of the natural system should be empirically considered *ceteris paribus* with respect to the hardware used (since the same procedure running on different hardware may lead to different execution times). This measure, however, should not be confused with algorithmic complexity times since, for example, two different exponential solutions, despite being equivalent from a computational complexity perspective, in practical applications, would still have a different execution time depending on the hardware on which they run. From an experimental perspective, this means that if the use of a more powerful hardware (leading to a consequent execution time closer to human performances) is driven by an intrinsic computational versatility of a particular modelling solution [e.g., we know that neural computation on Graphical Processing Units (GPUs) is a crucial feature of current large-scale connectionist model, while on the other hand, it is not similarly easily exploitable for symbolic AI computation], then such a solution would be preferable on the MCG scale with respect to an equivalent one that, however, is unable to exploit at its best the state-of-the-art hardware capabilities. As a consequence, the execution time requirement for the “performance match” criterion is a stronger constraint than the algorithmic equivalence based on time complexity.

6 For a complete comparison with such tests, and others, I refer to Lieto (2021), chapter 5.

7 This trajectory happens also during an intermediate period in which the monkeys, before learning to control remotely the cursor, continue to move their limbs.

8 In the case of brain controlled experiment by Carmena et al. (2003), the input managed by the neurons whose activity controlled the prosthesis were not those providing input to the animals’ biological arms, as the prosthesis was controlled by the activity of various frontoparietal neural ensembles acquired through a multielectrode brain–machine interface).
